# Clinical-epidemiological changes in patients with non-traumatic acute abdomen during the COVID-19 pandemic: a retrospective study

**DOI:** 10.1590/0100-6991e-20223303-en

**Published:** 2022-09-28

**Authors:** GUSTAVO RODRIGUES ALVES CASTRO, TIAGO AUGUSTO ZWIERZIKOWSKI, JOÃO GABRIEL DA SILVA LEMES, VALERIA MIDORI GUTOSKI YUKI, KAUANA OLIVEIRA GOUVEIA, CAMILA ROGINSKI-GUETTER

**Affiliations:** 1 - Hospital do Trabalhador, Serviço de Cirurgia Geral - Curitiba - PR - Brasil; 2 - Pontifícia Universidade Católica do Paraná - Curitiba - PR - Brasil; 3 - Johns Hopkins Bloomberg School of Public Health - Baltimore - Maryland - Estados Unidos

**Keywords:** Acute abdomen, COVID-19, Surgical Procedures of the Digestive System, Emergencies, Abdome Agudo, COVID-19, Procedimentos Cirúrgicos do Sistema Digestório, Emergências

## Abstract

**Objective:**

we intend to demonstrate the clinical alterations and the postoperative evolution in patients with acute abdomen non-traumatic in conservative or surgical treatment during the pandemic compared to a similar period in the last year.

**Method:**

a single-center retrospective study, including patients who received clinical-surgical treatment at Hospital do Trabalhador diagnosed with acute abdomen between March and August 2020 and a similar period in 2019.Variables studied ranged from demographic data to indices of social isolation.

**Results:**

515 patients were included, 291 received treatment in a pre-pandemic period and 224 during. There was not statistical difference in relation to comorbidities (p=0.0685), time to diagnosis and seeking medical help. No statistical differences were observed in terms of days of hospitalization (p = 0.4738) and ICU need (p=0.2320). Regarding in-hospital deaths, there was statistical relevance in the age above 60 years (p=0.002) and there were more deaths during the pandemic period (p=0.032). However, when we analyze the factors associated with the number of days until diagnosis by a physician, there was no statistical difference.

**Conclusion:**

the analyzed data showed that the pandemic period and age over 60 years were the variables that increased the odds ratio for the in-hospital death outcome. However, the length of stay, days in intensive care unit and postoperative surgical complications showed no significant difference.

## RESUMO

**Objetivo:**

pretendemos demonstrar as alterações clínicas e a evolução pós-operatória em pacientes com abdome agudo não traumático em tratamento conservador ou cirúrgico durante a pandemia em comparação a período semelhante no ano anterior.

**Método:**

estudo retrospectivo unicêntrico, incluindo pacientes que receberam tratamento clínico-cirúrgico no Hospital do Trabalhador com diagnóstico de abdome agudo entre março e agosto de 2020 e período semelhante em 2019. As variáveis estudadas variaram de dados demográficos a índices de isolamento social.

**Resultados:**

foram incluídos 515 pacientes, 291 receberam tratamento no período pré-pandemia e 224 na pandemia. Não houve diferença estatística em relação às comorbidades (p=0,0685), tempo para diagnóstico e procura de ajuda médica. Não foram observadas diferenças estatísticas quanto aos dias de internação (p=0,4738) e necessidade de UTI (p=0,2320). Em relação aos óbitos intra-hospitalares, observou-se relevância estatística na idade acima de 60 anos (p=0,002) e ocorreram mais óbitos no período da pandemia (p=0,032). Porém, quando analisamos os fatores associados ao número de dias até o diagnóstico por um médico, não houve diferença estatística.

**Conclusão:**

os dados analisados mostraram que o período de pandemia e a idade acima de 60 anos foram as variáveis que aumentaram a razão de chances para o desfecho óbito hospitalar. No entanto, o tempo de internação, dias na unidade de terapia intensiva e complicações cirúrgicas pós-operatórias, não apresentaram diferença significativa.

## INTRODUCTION

In late 2019, a novel coronavirus (SARS-CoV-2) was identified as a cause of pneumonia and severe acute respiratory syndrome. After rapid spread, on March 11, 2020, the World Health Organization (WHO) classified the situation as a global pandemic^1^. Since the first case of COVID-19, all health systems have adapted to this new dynamic, and new recommendations and protocols were developed^2^ ^,^ ^3^. Surgical departments were affected, recovery beds were transformed into ICU (intensive care unit) ones, elective procedures were postponed, and members of the surgical teams were allocated to reinforce the ICU^4^, certainly contributing to the decrease in the number of surgeries.

In this scenario, acute abdominal cases, conditions with potential complications and death in a short period of time, continue to appear^5^. Complications can present rates between 8.2 and 31.4%, and mortality is quite variable, being directly related to the underlying cause^6^ ^,^ ^7^. The burden on the health system caused by the COVID-19 pandemic results in difficult access and delay in the diagnosis and treatment of cases of acute abdomen, which can contribute to the increase in morbidity and mortality of diseases that require urgent surgical treatment.

Therefore, this study aims to assess possible changes in the clinical course and postoperative outcomes of patients with non-traumatic acute abdomen undergoing surgical or conservative treatment during the COVID-19 pandemic, when compared to a similar period prior to the pandemic.

## METHODS

This is a single-center, retrospective study developed under the approval of the Ethics Committee in Research Involving Human Beings of the Hospital do Trabalhador (CEPSH-SESA/HT), CAAE 33750120.7.0000.5225. Inclusion criteria were adult patients (>18 years) who received medical treatment at the Hospital do Trabalhador, in Curitiba, State of Paraná, Brazil, for a clinical-surgical condition of acute abdomen. Patients were identified by searching electronic medical records for ICD-10 codes compatible with diagnoses of acute abdomen, other abdominal pain, acute appendicitis, acute cholecystitis, acute pancreatitis, perforated peptic disease, acute diverticulitis, and hernia (femoral, inguinal and umbilical) with intestinal obstruction, under ICD’s R100, R104, K350, K551, K359, K810, K811, K818, K819, K850, K851, K852, K853, K858, K859, K271, K403, K404, and K420 codes, respectively. We included patients who met such criteria and who were seen between March to August 2019 and March to August 2020.

We excluded trauma patients, pediatric patients, individuals undergoing emergency surgery related to previous elective surgeries, and those with chronic abdominal pain (without concomitant acute complications), in addition to patients treated outside the pre-established preoperative period.

We set up two study groups according to the date of medical care. Patients seen between March 2020 and August 2020 constituted the group of patients seen during the COVID-19 pandemic. Another comparative group was formed by patients seen in a similar period, between March 2019 and August 2019, constituting the pre-COVID-19 pandemic group. Only patients who had respiratory symptoms or symptoms compatible with COVID-19 were tested for the identification of the new coronavirus.

Recorded variables included patient demographics, clinical-surgical diagnosis, time between symptoms onset and diagnosis, comorbidities (calculation of the Charlson Comorbidity Index, CCI), length of stay, in-hospital complications (ClavienDindo score), and surgical treatment. The CCI is one of the most used comorbidity indexes to predict mortality, identifying the present comorbidities and applying weights to these diseases, that is, mild (1-2), moderate (3-4), and severe (≥5)^8^. The ClavienDindo score was a standardized classification proposed in 1992 and revised in 2004, with severity levels based on the therapeutic intervention applied to the management of surgical complications^9^.

Indices of social isolation were also collected in the state where the hospital in question is located (Paraná), available on the public data platform “inloco”10. These data were obtained for the months of March to August 2020 (during the pandemic), with average indices calculated for each study month.

The collected data were then analyzed using the statistical software STATA v14.2^11^. For descriptive analysis, we expressed measures of central tendency and dispersion as mean and standard deviation (mean ± SD) for continuous variables with normal distribution and as medians, minimum and maximum values (median, minimummaximum) for those with non-normal distribution. Categorical variables were expressed as absolute and relative frequencies. For inferential statistical analysis, we compared groups using the unpaired Student’s t test for continuous dependent variables and the chisquare test for binary or categorical dependent variables. Finally, for the unpaired analysis of independent categorical variables, we used the KruskallWallis’s test. Multivariate logistic regression and linear regression were used to identify factors related to hospital death (categorical dependent variable) and days between onset of symptoms and seeking medical care (continuous dependent variable), respectively. The regression models and their parameters were developed based on a model with biological and epidemiological plausibility, as well as using the Akaike Information Criterion (AIC)^12^. A significance level of 5% was considered for this study.

## RESULTS

A total of 515 patients were included, with 291 attended in the pre-pandemic period and 224 in the COVID-19 pandemic period. The number of male patients was higher in both groups (Table 1). The age distribution was statistically different between the groups (p=0.036), though both showed a preponderance of non-elderly adults (<60 years).

**Table 1:** Distribution of 515 patients with acute abdomen according to clinical characteristics, divided into two groups, pre-pandemic and during the pandemic.

	Pre-COVID-19 pandemic (n=291)	During the COVID-19 pandemic (n=224)	p
Male n (%)	151 (51.89)	126 (56.25)	0.3250
Age n (%)			*0.036
<60	218 (74.91)	185 (82.59)	
≥60	73 (25.09)	39 (17.41)	
CCI (mean +/- SD)	1.21 (1.93)	0.92 (1.65)	0.0685
CCI % (average +/- SD)	88.22 (22.12)	90.01 (21.63)	0.3587
Previous abdominal surgery n (%)	93 (31.96)	82 (36.61)	0.2700
Days until diagnosis (mean +/- SD)	3.89 (6.11)	3.80 (12.56)	0.9108
Diagnosis n (%)			0.2200
Acute appendicitis	73 (25.17)	76 (34.86)	
Acute cholecystitis	74 (25.52)	43 (19.72)	
Acute pancreatitis	7 (2.41)	2 (0.92)	
Incarcerated/strangulated hernia	13 (4.48)	12 (5.50)	
Bowel obstruction	33 (11.38)	24 (11.01)	
Intestinal perforation	15 (5.17)	12 (5.50)	
Other	75 (25.86)	49 (22.48)	

ICC: Índice de Comorbidades de Charlson (1987)8; DP: desvio padrão.

There was no significant difference between the groups regarding previous comorbidities measured by the CCI (p=0.0685), as well as the presence of previous abdominal surgery (p=0.2700). There was also no difference in the time between the onset of symptoms and diagnosis when comparing the pre-pandemic (3.89 ± 6.11 days) and pandemic (3.80 ± 12.56 days) groups (p=0.9108).

The frequency of each diagnosis of acute abdomen among the patients included in this study was also similar between the groups (p=0.2200). Acute appendicitis and acute cholecystitis were the two most prevalent diagnoses in both groups.

Regarding hospital course (Table 2), the average of hospitalization days was similar between groups, 4.16 ± 5.30 days pre-pandemic and 4.50 ± 5.49 days during the pandemic. There was also no statistically significant difference between the groups regarding the use of antibiotic therapy, the need for a surgical approach, or the prevalence of different surgical techniques (open versus laparoscopic).

**Table 2:** Complications and hospital course pre- and during the pandemic.

	Pre-COVID-19 pandemic (n=291)	During the COVID-19 pandemic (n=224)	p
Length of hospital stay (mean +/- SD)	4.16 (5.30)	4.50 (5.49)	0.4738
Use of antibiotic therapy n (%)	108 (37.11)	99 (44.20)	0.1040
Days of antibiotic therapy (mean +/- SD)	5.59 (4.01)	5.54 (4.25)	0.9415
Need for surgery n (%)	195 (67.01)	149 (66.52)	0.9060
Open surgical technique n (%)	90 (42.65)	65 (42.48)	0.9740
Need for ICU n (%)	40 (13.75)	23 (10.27)	0.2320
ICU days (mean +/- SD)	6.63 (8.21)	7.87 (5.99)	0.5277
ClavienDindo n (%)			0.6960
Grade I	85 (29.21)	69 (30.80)	
Grade II	1 (0.34)	1 (0.45)	
Grade III A	28 (9.62)	26 (11.61)	
Grade III B	161 (55.33)	114 (50.89)	
Grade IV A	6 (2.06)	2 (0.89)	
Grade IV B	4 (1.37)	3 (1.34)	
In-hospital death n (%)	6 (2.06)	9 (4.02)	0.1910

SD: standard deviation; ICU: Intensive Care Unit.

As for the need for intensive care, the percentage of patients who required ICU was slightly lower during the pandemic, while the average length of stay in the ICU in days was slightly longer. However, these differences were not statistically significant.

Finally, there was also no difference between the groups regarding complications during hospitalization according to the Clavien-Dindo classification (p=0.6960). In both groups, grade III B was the most prevalent, characterizing surgical, endoscopic, or radiological intervention under general anesthesia. Six patients died in the hospital in the pre-pandemic period and nine during the pandemic, with no statistical difference.

We selected a logistic regression model using the independent variables sex, age, CCI, and group (pre-pandemic vs. pandemic). The dependent variable for this model was hospital death (Table 3). In the univariate analysis, the factors age ≥60 years (Odds Ratio 26.33, 95% CI 5.85118.57) and CCI (Odds Ratio 1.54, 95% CI 1.301.81) were associated with in-hospital death (both p<0.001). After adjusting for these covariates in the multivariate analysis, age ≥60 (Odds Ratio 13.96, 95% CI 2.5775.91, p=0.002) and CCI remained statistically associated with in-hospital death (Odds Ratio 1.30, 95% CI 1.031.63, p=0.029). In addition, after adjusting for covariates, being seen during the pandemic proved to be a factor associated with in-hospital death (Odds Ratio 3.54, 95% CI 1.1112.27, p=0.032).

**Table 3:** Logistic regression, factors associated with in-hospital death.

	Unadjusted OR	p	Adjusted OR	p
Sex				
Female	0.77 (0.27 - 2.20)	0.625	0.88 (0.28 - 2.75)	0.8260
Age				
≥60	26.33 (5.85 - 118.57)	*<0.001	13.96 (2.57 - 75.91)	*0.002
CCI	1.54 (1.30 - 1.81)	*<0.001	1.30 (1.03 - 1.63)	*0.029
Being seen during the COVID pandemic	1.99 (0.70 - 5.67)	0.199	3.54 (1.11 - 11.27)	*0.032

CCI: Charlson Comorbidity Index (1987)^8^.

We then built a linear regression model using the independent variables sex, age, CCI, and social isolation index per month (during the pre-pandemic period). The dependent variable of this model was the number of days between the onset of symptoms and diagnosis (Table 4). None of the variables was associated with delay to diagnosis, either in the univariate analysis or after adjusting for covariates.

**Table 4:** Linear regression, factors associated with the number of days until diagnosis.

	Beta coefficient (unadjusted)	p	Beta coefficient (adjusted)	p
Sex				
Female	0.01 (-1.64 - 1.65)	0.992	-1.15 (-4.51 - 2.22)	0.5030
Age				
≥60	-1.63 (-3.61 - 0.36)	0.108	-1.95 (-7.82 - 3.91)	0.5120
CCI	-0.22 (-0.67 - 0.23)	0.334	0.40 (-0.95 - 1.75)	0.5560
Social isolation index (5% groups)	-2.11 (-5.34 - 1.11)	0.198	-2.27 (-5.52 - 0.99)	0.1710

CCI: Charlson Comorbidity Index (1987)^8^.

## DISCUSSION

With the evolution of the COVID-19 pandemic, several aspects of care for acute abdominal disorders were adapted to optimize available resources. International surgical societies have published new protocols with guidelines on surgical treatment versus conservative treatment in cases of acute abdomen during the pandemic^13^ ^-^ ^15^.

The pandemic has brought unprecedented challenges to health systems^16^, but even after more than 24 months, there are still few reports of its effect on the care of acute abdomen cases. Some authors have reported delays in medical care for the pediatric population^17^ ^,^ ^18^, which may be related to parents’ fear of submitting their children to a hospital environment during the pandemic^19^. In addition, the profile of patients admitted to a hospital environment has changed^20^, reducing the number of surgical patients in several hospitals5. Perhaps due to more restrictive measures, there was a decrease in the number of patients hospitalized for non-traumatic causes in South Africa^20^. Other international studies show a reduction in emergency admissions, such as an Italian study^21^ and a North American one^22^. A study from New Zealand, for example, found a reduction of 26%^23^. In our study, despite the decrease in the absolute number of patients, this difference was not statistically relevant.

During the pandemic, patients under 60 years of age without previous abdominal surgery predominated, with an average of 3.80 days from the onset of symptoms to diagnosis. These data are statistically similar to the pre-pandemic period. As for diagnoses, appendicitis and acute cholecystitis predominated in the two periods studied. Thus, we observed that patients continued to seek medical help, despite the restrictions imposed by social isolation. The reasons may be the maintenance of access to emergency rooms even with the increase in hospital occupancy and to the milder isolation restrictions applied in the institution’s city and state, or even to the communication carried out by the media in general and through medical channels, guiding the population.

When evaluating data on length of stay, days in the intensive care unit, and number of deaths, we found no significant difference between the groups. Such findings are similar to data on mortality and length of stay found in the literature, in which there was also no statistical difference during the pandemic period^23^.

We used the Clavien-Dindo classification of surgical complications to verify the presence and severity of postoperative complications, and we did not obtain statistical difference between the groups, the III B classification being the most prevalent in both, which characterizes the need for surgical, endoscopic, or radiological intervention under general anesthesia.

When we turned to the literature to make a comparison between the period before and during the pandemic, British studies found similarities and good results in surgeries for acute appendicitis^24^ ^,^ ^25^, although some trials showed higher mortality in patients undergoing surgical procedures during the pandemic^26^.

However, when using logistic regression models, as shown in Tables 3 and 4, we obtained interesting findings. Table 3 specifies the outcome of death, both in univariate and multivariate analysis, through which we observed statistical significance. In the multivariate analysis, for example, being male or female did not interfere with death, but being over 60 years old displayed an odds ratio of 13.96 times more in-hospital deaths, and being treated during the pandemic had a mortality risk 3.54 times greater than patients seen in the pre-pandemic period. This last finding brings an important reflection: if there was no statistical difference between the time until diagnosis and there were no differences in postoperative complications, as seen in the data in Tables 1 and 2, this difference could be related to in-hospital difficulties due to overcrowding and scarcity of resources. Apart from the first two months of the pandemic, the team always worked with limited capacity, due to the demand from COVID, trauma, and non-traumatic acute abdomen, but we did not find detailed monthly data on scarcity of resources and intra-hospital difficulties.

In Table 4, we evaluated the variables age, sex, and social isolation index with the number of days from the onset of symptoms to hospital care and we found no association between the variables and the outcome. We could infer that social isolation did not delay the search for hospital care, as the elderly continued to seek the emergency room during the pandemic.

Our work has some limitations inherent to the study design, as it is a non-randomized study in only one institution and with a limited number of patients. However, it is valid as a portrait of the possible effects of the pandemic on the care of acute abdominal disorders in Brazil and as a support for future work to be carried out with larger samples and with more participating institutions.

## CONCLUSIONS

During the pandemic, patients over 60 years old had a higher risk of complications and death than in the pre-pandemic period. However, we did not find changes regarding the time of clinical history and diagnosis, nor there were any changes regarding the length of stay, days in the intensive care unit, and postoperative surgical complications when comparing the two periods studied.
